# CDK4/6 inhibition enhances pulmonary inflammatory infiltration in bleomycin-induced lung fibrosis

**DOI:** 10.1186/s12931-020-01433-w

**Published:** 2020-07-02

**Authors:** Anna Birnhuber, Bakytbek Egemnazarov, Valentina Biasin, Ehsan Bonyadi Rad, Malgorzata Wygrecka, Horst Olschewski, Grazyna Kwapiszewska, Leigh M. Marsh

**Affiliations:** 1 Ludwig Boltzmann Institute for Lung Vascular Research, Neue Stiftingtalstrasse 6/VI, 8010 Graz, Austria; 2 Division of Endocrinology and Diabetology; Department of Internal Medicine, Medical University of Graz, Graz, Austria; 3 Department of Biochemistry, Universities of Giessen and Marburg Lung Center, Member of the German Center for Lung Research, Giessen, Germany; 4 Division of Pulmonology, Department of Internal Medicine, Medical University of Graz, Graz, Austria; 5 Otto Loewi Research Center, Medical University of Graz, Graz, Austria

**Keywords:** CDK4/6 inhibition, Palbociclib, Pulmonary inflammation, Interstitial lung disease

## Abstract

Inhibitors of cyclin-dependent kinases 4/6 (CDK4/6) block cell cycle progression and are commonly used for treatment of several forms of cancer. Due to their anti-proliferative mode of action, we hypothesized that palbociclib could attenuate the development of bleomycin-induced lung fibrosis. In a preclinical setting, mice were treated with bleomycin and then co-treated with or without palbociclib. Lung function, collagen deposition and pulmonary inflammation were analysed after 14 days.

Bleomycin treatment led to an increase of pulmonary fibrosis and inflammation, and concomitant decline of lung function. Palbociclib treatment significantly decreased collagen deposition in the lung after bleomycin treatment, but did not ameliorate lung function. Importantly, palbociclib augmented inflammatory cell recruitment (including macrophages and T cells) in the bronchoalveolar lavage fluid.

This study supports the recent alert from the Food and Drug Administration (FDA) that use of CDK4/6 inhibitors, such as palbociclib, may have severe pulmonary adverse effects. Our study showing heightened pulmonary inflammation following palbociclib treatment highlights the risk of severe inflammatory adverse effects in the lung. This is of special interest in patients with known pulmonary risk factors and emphasizes the need of careful monitoring all patients treated with CDK4/6 inhibitors for signs of lung inflammation.

**To the Editor:**

A recent Food and Drug Administration (FDA) warning has alerted the respiratory community that the use of cyclin-dependent kinases 4 and 6 (CDK4/6) inhibitors may lead to severe pulmonary inflammation. Data from different clinical trials and post-market databases has revealed cases of severe interstitial lung disease and pneumonitis, including fatalities, in one to 3 % of patients following treatment with CDK4/6 inhibitors [1]. Several case reports highlighted severe pneumonitis in the absence of any bacterial, viral, fungal infection, indicating drug-induced pulmonary toxicity following CDK4/6 inhibition [2, 3] During normal cell proliferation CDK4/6 binds cyclin D1, which then hyperphosphorylates the retinoblastoma protein (Rb) leading to the release and activation of the transcription factor E2F1, which in turn activates genes important for cell cycle progression. Palbociclib and other CDK4/6 inhibitors, such as abemaciclib and ribociclib, block this process by preventing formation of the CDK4/6-cyclin D1 complex, leading to cell cycle arrest at the G1/S checkpoint and thereby preventing tumor cell growth [4]. As several CDK4/6 inhibitors are FDA-approved or are in phase II clinical trials for the treatment of diverse forms of cancer, the number of patients at risk for pulmonary adverse effects is a very relevant concern [5].

Data from our laboratory reinforces this need to carefully evaluate pulmonary inflammatory side effects following CDK4/6 inhibition. In a preclinical experimental setting we investigated whether blockage of cell proliferation prevented bleomycin-induced lung fibrosis. Bleomycin-treated mice were co-treated with palbociclib (PD 0332991, 150 mg/kg/day) in a preventive fashion (Fig. 1a), as described previously [6–8].

**Fig. 1:**
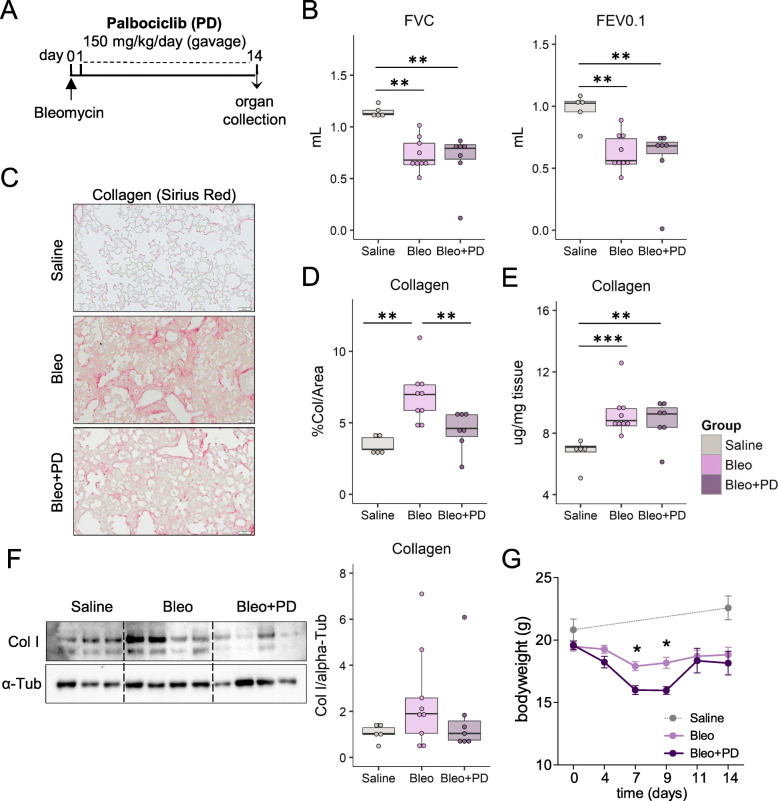
Palbociclib decreases collagen deposition but does not improve lung function in the bleomycin-mouse model. **a** Schematic representation of palbociclib treatment in bleomycin-induced lung fibrosis. Lung injury was induced by intratracheal bleomycin (Bleo) instillation (0.8 units/g bodyweight) at day 0, followed by daily oral gavage with 150 mg/kg bodyweight palbociclib (PD 0332991) in a subgroup of mice (Bleo+PD), starting from day 1. Lung function measurements and organ collection were performed 14 days post bleomycin. Control animals received intratracheal saline. **b** Lung function measurements were performed using a flexiVent FX1 (Scireq) system. FVC: forced vital capacity, FEV0.1: forced expiratory volume after 0.1 s; Kruskal Wallis test; ** *p* < 0.01. Collagen content of the lung was measured on Sirius Red stained lung sections of the entire left lung lobe (**c**) with semi-automated quantification (**d**) using the Visiopharm integrated software. **e** Hydroxyproline measuements were performed on tissue homogenates from right lung pieces. **f** Representative immunoblot of collagen I in lung homogenates of saline, bleomycin, and bleomacin+palbociclib treated mice. α-tubulin served as a loading control. **g** Bodyweight curves of mice following bleomycin-induced lung injury. *n* = 4–6, data are shown as mean ± SEM. Bleo and Bleo+PD groups were compared by two-way ANOVA with Bonferroni post-test; *p < 0.05, **p < 0.01, ***p < 0.001

As characteristic of this model, bleomycin decreased lung function, including a reduced forced vital capacity (FVC) and forced expiratory volume (FEV0.1; Fig. 1b), and increased collagen deposition in the lung (Fig. 1c-f). After 2 weeks of treatment with palbociclib, collagen deposition was reduced as indicated by quantification using image analysis of Sirius Red staining of the whole left lung lobe (Fig. 1c, d). This was supported by western blot analysis of lung tissue homogenates (Fig. 1f), whereas hydroxyproline measurements showed no alteration between the bleomycin-treated groups with or without palbociclib treatment (Fig. 1e). Lung function was also not improved (Fig. 1b). Furthermore, palbociclib treated mice showed a greater loss of bodyweight compared to mice treated with bleomycin only, suggesting more deleterious effects. This difference was most evident during the acute inflammatory phase of bleomycin treatment around day 7–9 [7], and less pronounced thereafter (Fig. 1e).

A potential explanation of this adverse effect of palbociclib, is the amplified inflammatory cell recruitment in the bronchoalveolar lavage (BAL) of bleomycin-treated mice (Fig. 2a). In depth flow cytometry analysis of the inflammatory profile showed that palbociclib significantly increased levels of monocyte-derived and interstitial macrophages, dendritic cells, neutrophils, γδTCR+ and CD8+ effector T-cells (Fig. 2b, c). While, the levels of alveolar macrophages, eosinophils, B-cells and CD4+ T-helper cells were unchanged (Fig. 2b, c). Protein levels of neutrophil elastase (Elane) and myeloid peroxidase (MPO) activity in the bronchoalveolar lavage fluid were almost undetectable in saline-treated control mice and increased upon bleomycin treatment. Interestingly, co-treatment with palbociclib did not alter neutrophil elastase levels (Fig. 2d), or MPO activity (Fig. 2e). A possible explanation of these apparently opposing results is a recent paper by Amulic et al., which demonstrated that CDK4/6 activation is needed for extracellular trap (NET) formation and concomitant elastase release [9]. Therefore, one could speculate that the use of CDK4/6 inhibitors such as palbociclib could dampen neutrophil activation. However, the effects of palbociclib on the activation of other inflammatory cell types is currently unknown.

**Fig. 2:**
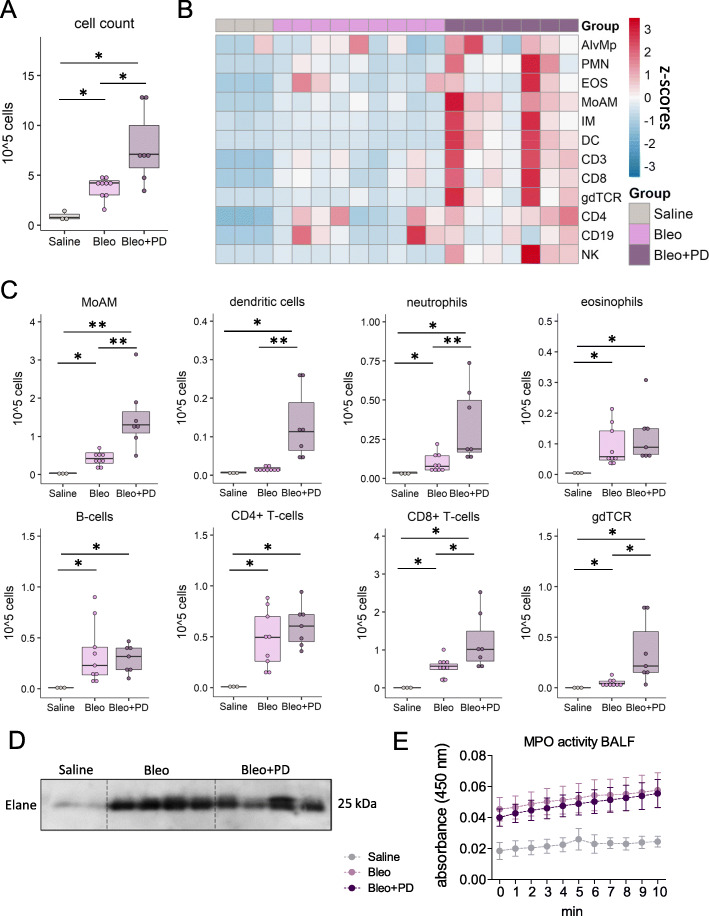
Palbociclib promotes lung inflammation following bleomycin challenge. **a** Total cell count in the bronchoalveolar lavage (BAL) of control mice (Saline) and mice with bleomycin-induced lung injury with (Bleo+PD) and without palbociclib (Bleo) treatment. Samples were collected 14 days after intratracheal bleomycin or saline instillation. The lung was lavaged with 1 ml of PBS supplemented with protease inhibitors. **b** Heatmap representation of the relative proportion of inflammatory cell populations in the BAL. z-scores are shown. **c** Absolute numbers of inflammatory cells in the BAL fluid of control and bleomycin-treated mice with and without palbociclib. MoAM: monocyte-derived macrophages; gdTCR: γδ T-cells. **d** Protein levels of neutrophil elastase (Elane) were determined in cell-free bronchoalveolar lavage fluid. **e** Activity of myeloid peroxidase (MPO) was measured by reduction of hydrogen peroxide using o-dianisidine as hydrogen donor and determining optical density at 450 nm (*n* = 2–6)

While palbociclib decreased collagen deposition, it increased inflammatory infiltration. These findings raise the question whether a certain amount or specific type of inflammation might ameliorate lung fibrosis for example by increased expression of collagen degrading enzymes, such as matrix metalloproteinases, or alternatively whether there may be a complete disconnect between inflammation and fibrosis. As a net effect of these beneficial and detrimental alterations, lung function was unchanged. Nevertheless, the elevation of pulmonary inflammation observed in this study and in clinical settings [2, 3], raises significant concerns regarding the putative fatal lung inflammation in patients treated with CDK4/6 inhibitors.

Mechanistically, the elevated levels of inflammatory cells observed in the bleomycin model as well as in cancer patients could be a consequence of the palbociclib-induced cell cycle arrest with the consequence of cellular senescence. Senescent cells (e.g. fibroblasts or epithelial cells) can promote a striking increase of inflammatory cytokines, growth factors and extracellular matrix (ECM) modulating proteins, a phenomenon called “senescence associated secretory phenotype” (SASP) [10]. Several studies have indicated that cellular senescence has an important impact on the development of pulmonary fibrosis. Isolated fibroblasts from idiopathic pulmonary fibrosis (IPF) lung tissue showed increased cellular senescence, including expression of p16 and p21, telomere shortening and mitochondrial dysfunction, together with a pro-fibrotic secretome, when compared to fibroblasts isolated from age-matched healthy lung tissue [11, 12]. Similarly, bleomycin-induced lung fibrosis increased senescence in murine lungs and ablation of senescent fibroblasts through genetic or pharmacological interventions recovered pulmonary function [13, 14]. While the effects observed in our study may be due to palbociclib-induced cellular senescence, causing SASP and therefore, enhanced inflammatory cell recruitment, detailed answers on the underlying mechanisms require further studies. For example, we cannot exclude possible synergistic effects of bleomycin and palbociclib, or whether a therapeutic approach with shorter exposure to palbociclib may maintain its beneficial effects while simultaneously avoiding excessive inflammatory infiltration and hence pulmonary toxicity.

## Conclusion

Although our study is limited by a lack of conclusive mechanisms, it supports the current FDA warning and highlights the danger of severe inflammatory adverse effects in the lung, especially in patients with known pulmonary risk factors. It further emphasizes the need to carefully monitor all treated patients for signs of pulmonary inflammation and to refrain from using this treatment in patients with interstitial lung disease, where even mild exacerbations could be fatal.

## Data Availability

The datasets used and/or analysed during the current study are available from the corresponding author on reasonable request.

## Abbreviations

FDA: Food and drug administration
CDK4/6: Cyclin-dependent kinases 4 and 6
Rb: Retinoblastoma protein
FEV0.1: Forced expiratory volume after 0.1 s
BAL: Bronchoalveolar lavage
FACS: Fluorescence activated cell sorting
γδTCR: gamma-delta T-cell receptor
CD: Cluster of differentiation
ECM: Extracellular matrix
SASP: Senescence-associated secretory phenotype
IPF: Idiopathic pulmonary fibrosis
NET: Neutrophil extracellular trap
